# Progression in Near-Infrared Fluorescence Imaging Technology for Lung Cancer Management

**DOI:** 10.3390/bios14100501

**Published:** 2024-10-14

**Authors:** Xinglong Chen, Yuning Li, Jialin Su, Lemeng Zhang, Hongwen Liu

**Affiliations:** 1 Thoracic Medicine Department 1, The Affiliated Cancer Hospital of Xiangya School of Medicine, Central South University/Hunan Cancer Hospital, Changsha 410013, China; chenxl@mail.hnust.edu.cn (X.C.); liyuning@mail.hnust.edu.cn (Y.L.); 22010901003@mail.hnust.edu.cn (J.S.); 2 School of Life and Health Sciences, Hunan University of Science and Technology, Xiangtan 411201, China; 3 College of Chemistry and Chemical Engineering, Hunan Normal University, Changsha 410081, China; liuhongwen@hnu.edu.cn

**Keywords:** lung cancer, diagnosis, treatment, near-infrared fluorescence, in vivo imaging

## Abstract

Lung cancer is a major threat to human health and a leading cause of death. Accurate localization of tumors in vivo is crucial for subsequent treatment. In recent years, fluorescent imaging technology has become a focal point in tumor diagnosis and treatment due to its high sensitivity, strong selectivity, non-invasiveness, and multifunctionality. Molecular probes-based fluorescent imaging not only enables real-time in vivo imaging through fluorescence signals but also integrates therapeutic functions, drug screening, and efficacy monitoring to facilitate comprehensive diagnosis and treatment. Among them, near-infrared (NIR) fluorescence imaging is particularly prominent due to its improved in vivo imaging effect. This trend toward multifunctionality is a significant aspect of the future advancement of fluorescent imaging technology. In the past years, great progress has been made in the field of NIR fluorescence imaging for lung cancer management, as well as the emergence of new problems and challenges. This paper generally summarizes the application of NIR fluorescence imaging technology in these areas in the past five years, including the design, detection principles, and clinical applications, with the aim of advancing more efficient NIR fluorescence imaging technologies to enhance the accuracy of tumor diagnosis and treatment.

## 1. Introduction

Lung cancer remains the leading cause of cancer-related deaths worldwide. In 2022, 1,918,030 new cancer cases were reported in the United States, with lung cancer accounting for 609,360 cases, making it the leading cause of cancer death. Although survival rates have improved significantly, largely due to advances in early diagnosis and treatment methods, the early detection of lung cancer remains crucial for improving cure rates and overall prognosis [1,2,3,4,5,6]. Nevertheless, the challenge of early detection remains formidable. Existing methods have limitations, such as low sensitivity and poor specificity, complicating early diagnosis and hindering the effective in vivo dynamic observation of tumors. These limitations underscore the urgent need for the development of new, more accurate detection techniques [7,8,9,10]. Recent advances in medical imaging, particularly in fluorescence imaging techniques, have led to significant progress in tumor management. Notably, NIR fluorescence imaging has garnered significant attention due to its superior properties, including longer emission wavelengths, deeper tissue penetration, and enhanced contrast in biological environments compared to other fluorescence types. These features enhance imaging clarity and accuracy, positioning NIR fluorescence imaging as a promising tool for early tumor detection and monitoring [11,12,13,14,15,16,17,18].

**Scheme 1:**
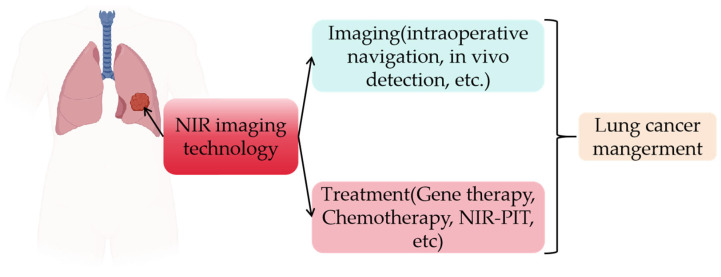
Current application direction of NIR imaging technology in lung cancer management.

Scientists have recently made significant progress in NIR fluorescence imaging research for lung cancer (Scheme 1). However, despite these advances, challenges remain, including the optimization of probe sensitivity and specificity and the improvement of technology applicability in clinical settings. Additionally, enhancing depth imaging capabilities for a more accurate understanding of lung cancer progression is crucial. These are just some of the current challenges. This review proposes unique solutions to these challenges, aiming to effectively address them. Given the high incidence and mortality of lung cancer, along with poor post-treatment prognosis, NIR fluorescence imaging technology offers new hope for addressing these clinical challenges. Additionally, fluorescence detection integrated with diagnosis and treatment has garnered significant attention. In recent years, numerous NIR fluorescence imaging technologies have been applied to tumor detection and treatment, with some reviews summarizing fluorescent probes used for tumor imaging. However, even fewer reviews focus on the subdivision of NIR fluorescence imaging technology concerning the imaging, treatment, and prognosis of lung cancer. For example, some reviews do not focus exclusively on lung cancer but cover various cancers; others, while focused on lung cancer, do not prioritize NIR fluorescence technology, which offers distinct advantages. While some reviews meet these criteria, their content is limited to NIR fluorescent probes and neglects other detection platforms that utilize the principles of NIR fluorescence. Detection platforms based on these principles are rarely addressed. Therefore, this review will summarize NIR fluorescence imaging technology from multiple perspectives, complementing existing reviews. This includes a preliminary summary of clinical translation, an overview of imaging functions, and explanations of the various therapies associated with the probes, including their principles and conditions. Ultimately, this review aims to comprehensively present detailed information on these technologies, providing researchers with accurate and comprehensive references [11,12,13,14,15,16,17,18].

This review summarizes the latest research progress in NIR fluorescence imaging for lung cancer. By summarizing the current research status, we focus not only on imaging but also on various therapeutic functions (such as photodynamic/photothermal therapy, gene therapy, etc.). Additionally, the review highlights the research results and challenges faced and briefly summarizes some clinical application data, aiming to provide readers with a broader perspective, explore potential future development directions, and offer valuable insights and guidance for researchers and clinicians in the field. The innovation of this review lies in its summary of recent advances and critical analysis of current challenges, which is more comprehensive, with a focus on lung cancer and NIR fluorescence technology, thereby contributing to the future diagnosis and treatment of lung cancer.

## 2. Principle and Type of Fluorescent Probe

**Figure 1:**
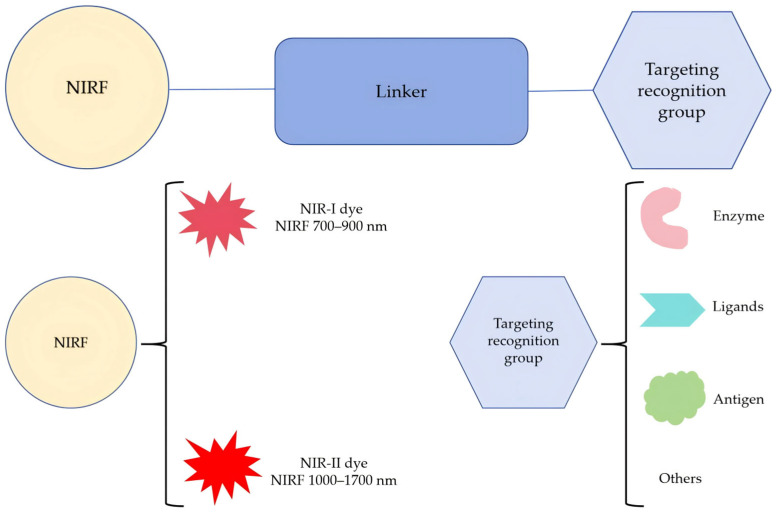
Common design strategies and components of NIR fluorescent probes.

Figure 1 shows half of the design strategies and components of a fluorescent probe.

### 2.1. Action Principle of NIR Fluorescent Probe

The basic working principle of fluorescent probes involves utilizing the optical properties of fluorescent groups to activate fluorescent signals through their binding and interaction with the target object, thereby enabling the in vivo imaging of tumors. Initially, researchers consider the target of the fluorescent probe during the design phase. The target typically binds to a specific part of the fluorescent probe, resulting in a change in the probe’s molecular structure. Following this alteration in molecular structure, the luminescence mechanism chosen by the researchers—such as photoinduced electron transfer (PET), Intramolecular Charge Transfer (ICT), Twisted Intramolecular Charge Transfer (TICT), or Förster Resonance Energy Transfer (FRET)—is activated to generate fluorescence. Furthermore, researchers utilize various designs to enhance the targeting capabilities of fluorescent probes, allowing them to accumulate in specific tumor sites. At this stage, imaging instruments can detect the specific wavelengths of fluorescence emitted by various fluorescent groups. By identifying the target object and binding to it, the fluorescent probe specifically targets the tumor site. Upon activation, the fluorescent group emits a signal at a specific wavelength. Detecting this signal enables the determination of the tumor’s existence, location, size, and other pertinent information, facilitating early diagnosis and ongoing monitoring of the tumor. Due to the emission wavelengths of NIR fluorescent probes, categorized as NIR-I (700–900 nm) and NIR-II (1000–1700 nm), the longer emission wavelengths demonstrate superior tissue penetration and an improved signal-to-background ratio compared to other fluorescent probes, establishing them as effective tools in lung cancer diagnosis and treatment.

Concurrently, after establishing the fundamental imaging function, additional functionalities, such as photothermal therapy, are usually integrated into fluorescent probes. This involves using light of a specific wavelength to irradiate tumor tissues, often employing photosensitizers to enhance light absorption. When light irradiates tumor cells, photosensitizers absorb light energy and convert it into heat, resulting in an increase in temperature that induces either apoptosis or necrosis. Photodynamic therapy involves the combination of photosensitizers with light exposure. Once photosensitizers accumulate in tumor tissues, they are irradiated with light of a specific wavelength, generating reactive oxygen species. These reactive oxygen species can damage the membranes and DNA of tumor cells, resulting in cell death and localized tissue destruction. Immunogenic cell death therapy induces immunogenic cell death in tumor cells, prompting the immune system to recognize and attack them. This therapy can be combined with chemotherapy, radiotherapy, or immunotherapy to activate tumor cells, leading to the release of specific signaling molecules such as HMGB1, ATP, and heat shock proteins. These molecules stimulate the activation and recruitment of immune cells, thereby enhancing the immune response to tumors. The integration of these functions represents a significant trend in the development of fluorescent probe technology, achieving a synergistic effect while facilitating integrated diagnosis, treatment, and prognosis.

### 2.2. Types of NIR Fluorescent Probes

Fluorescent probes can be generally categorized into three types based on their targets: (i) Tumor antigen probes, such as those that respond to carcinoembryonic antigen (CEA) and cytokeratin 19 fragment (CYFRA21-1); (ii) Tumor-related enzyme probes, including those that respond to neutrophil elastase (NE), aminopeptidase N (APN), β-galactosidase (β-gal), and neuron-specific enolase (NSE); (iii) Membrane receptor protein fluorescent probes, such as those that target epidermal growth factor receptor (EGFR) and anaplastic lymphoma kinase (ALK). Additionally, there are probes designed to assess the physical properties of the cell environment, such as those responding to pH, polarity, and viscosity.

## 3. Lung Cancer-Associated NIR Fluorescent Probes

Most enzymes and receptors on the surface of lung cancer cell membranes are proteins, and their counterparts may be glycoproteins. Additionally, there are numerous active substances in biological systems, such as reactive oxygen species, reactive nitrogen species, and reactive sulfur species. Under normal conditions, the activity and expression of these substances are relatively stable. However, when cells or tissues become diseased, the expression and activity of certain substances can become abnormally elevated. Current research indicates that the abnormal increase in the activity and expression of these substances is often associated with tumors [19,20,21,22,23,24,25,26].

NIR fluorescent probes developed from these substances can be internalized by tumor cells or their organelles through various mechanisms. For instance, certain probe molecules possess a small molecular weight and lipophilicity, allowing them to diffuse freely through the phospholipid bilayer of the cell membrane via passive diffusion. Additionally, some fluorescent probes are designed with targeting sites, which can bind tightly to the cell surface or utilize transport proteins on the cell membrane to enrich them on the cell surface or enter the cell through active transport mechanisms. Moreover, recent research studies have focused on enveloping fluorescent probe molecules within biological membranes, particularly for larger probes or those with specific surface functional groups. This design strategy enables the introduction of fluorescent probes into cells via endocytosis. Furthermore, some fluorescent probes, assembled into nanoparticles or delivered by carriers, can release their contents into cells through membrane fusion. Notably, imaging does not necessarily require fluorescent probe molecules to enter the cells. Overall, the imaging approach of fluorescent probes at the cellular level can be appropriately tailored based on the type of probe and the tumor characteristics. The NIR fluorescent probes discussed in this article primarily utilize the aforementioned mechanisms to facilitate lung cancer imaging.

### 3.1. Enzyme-Activatable Fluorescent Probes

**Figure 2:**
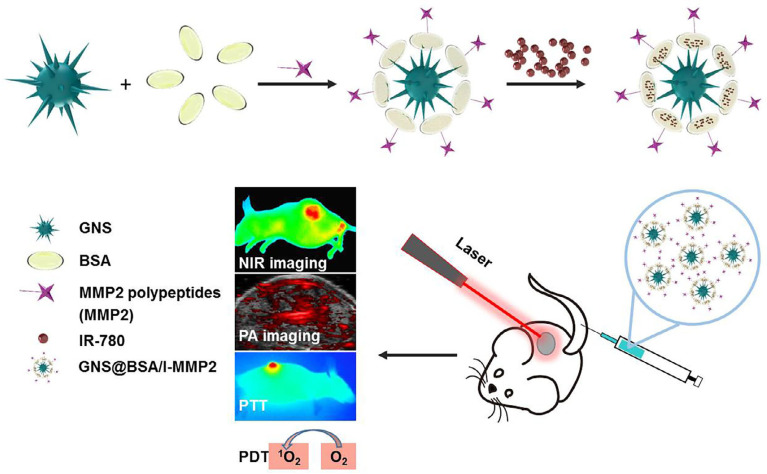
The construction process and application diagram of GNS@BSA/I-MMP2. Reproduced from [30] with permission from the Royal Society of Chemistry, Copyright 2019.

Matrix metalloproteinases (MMPs) can degrade the extracellular matrix and induce the vascularization of tumor tissue, enhancing its metastatic potential. Additionally, studies have shown that MMP-2 expression is increased in various cancers, including lung cancer, and is closely associated with tumor cell growth and migration [27,28,29]. In 2019, Xia et al. reported the GNS@BSA/I-MMP2 (Figure 2) multifunctional fluorescent probe, which responds to MMP-2 in vivo and effectively induces tumor cell apoptosis through enhanced photothermal and photodynamic therapy [30]. The researchers first synthesized gold nanoparticles (GNSs) and modified them with bovine serum albumin (BSA) to create GNS@BSA. They then further modified GNS@BSA with MMP-2-responsive peptides to obtain GNS@BSA-MMP2 nanoparticles, followed by the loading of the NIR fluorescent dye IR-780 iodide onto GNS@BSA-MMP2 nanoparticles through physical adsorption, resulting in a complete probe. The probe successfully performed photoacoustic imaging of tumors at different time points in A549 cell (a human non-small cell lung cancer cell line) tumor model mice, and the results showed that the probe can effectively kill tumor cells in vivo.

**Figure 3:**
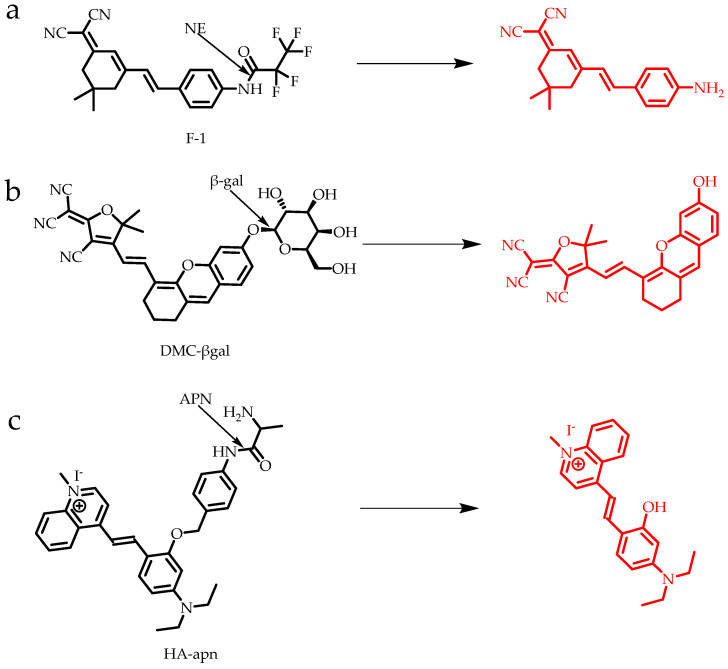
(**a**) Structure of F-1; (**b**) Structure of DMC-βgal; (**c**) Structure of HA-apn, and the effect of enzymes on their fluorescence activities. NE: neutrophil elastase; β-gal: β-galactosidase; APN: aminopeptidase N.

Neutrophil elastase (NE) is an important serine hydrolase primarily produced by neutrophils. It plays various biological roles in the human body and is significant in the occurrence and development of cancers, such as lung cancer. Therefore, accurately detecting NE activity is crucial for understanding its biological function, diagnosing related diseases, and monitoring disease progression [31,32,33,34]. In 2021, Zhang et al. employed a fluorescent probe named F-1 (Figure 3a) to image NE in A549 cells [35]. The team synthesized the fluorophore (E)-2-(3-(4-aminostyryl)-5,5-dimethylcyclohexam-2-en-1-ylidene)malononitrile (TMN-NH_2_) using their previously reported method. The probe was then conjugated with 1,1,1,2,2-pentafluoro-2-(perfluoroethoxy)ethane, a structure similar to the natural substrate of NE, which can be cleaved by NE to release the fluoroethoxy group and activate fluorescence. The experimental results confirmed that F-1 exhibits excellent imaging capabilities in A549 cells.

β-galactosidase (β-gal) is a glycoside hydrolase that catalyzes the hydrolysis of the β-galactoside bond, converting β-galactose into galactose and glucose. Its expression is elevated in certain cancers. Studies have shown that increased β-gal activity may be associated with tumor formation, proliferation, and metastasis [36,37,38]. In 2022, Li et al. introduced an NIR fluorescence-emitting dicyanomethylenedihydrofuran derivative (DMC-OH) into the β-galactose substrate to synthesize an ultrasensitive β-gal fluorescent probe, DMC-βgal (Figure 3b), with a minimum detectable concentration as low as 0.298 U/L [39]. This probe differentiated five different tumor cell lines, including SKOV-3 (a human ovarian cancer cell line), HepG2 (a human liver cancer cell line), HCT-116 (a human colon cancer cell line), A549, and MCF-7 (a human breast cancer cell line), based on differences in β-gal enzyme activity. Additionally, the activity of β-gal enzyme was also successfully detected in animal experiments on A549 tumor models, indicating that DMC-βgal is a highly sensitive, specific, and low-toxicity in vivo NIR fluorescent probe.

Aminopeptidase N (APN), also referred to as CD13 or leucine aminopeptidase, is a membrane-bound zinc exopeptidase that is widely expressed on the surface of mammalian cells. It is involved in various physiological and pathological processes and plays a significant role in cancer, inflammation, and the immune response [40,41]. In 2024, Dong et al. developed the fluorescent probe HA-apn (Figure 3c), which responds to APN to facilitate the rapid diagnosis of lung cancer [42]. The team initially synthesized p-aminobenzyl alcohol, followed by the synthesis of the fluorescent group HA. Following a series of reactions, the two components generated a fluorescent group containing p-aminobenzyl alcohol substituents. Finally, L-alanine residues were incorporated into this new structure to yield a complete probe. In cellular experiments, the researchers utilized the probe to successfully distinguish four tumor cell lines—A549, HeLa (a human cervical cancer cell line), TPC-1 (A human papillary thyroid carcinoma cell line), and HepG2—based on differences in APN activity. The team also monitored pronounced fluorescence signals in an orthotopic lung cancer mouse model by atomizing the probe, reaching a peak after 60 min.

**Figure 4:**
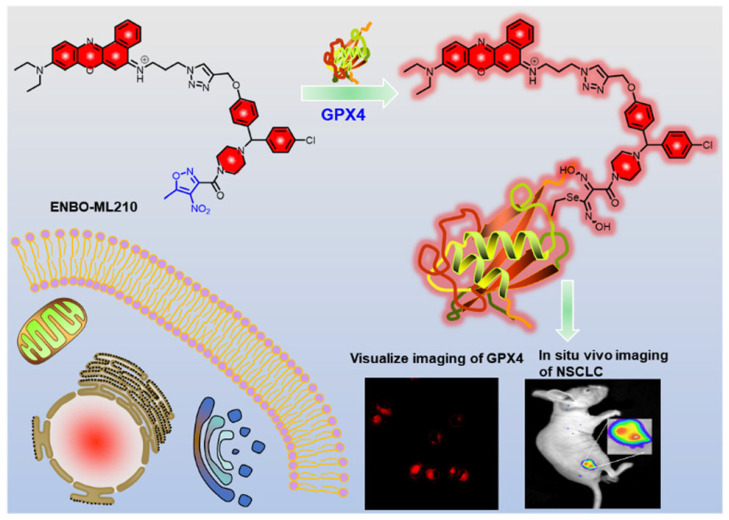
Structure of ENBO-ML210 and the influence of GPX4 on the fluorescence activation process and the in vivo imaging effect in NSCLC tumor model mice. Reproduced from [43] with permission from the Royal Society of Chemistry, Copyright 2023.

Glutathione peroxidase 4 (GPX4) is an essential antioxidant enzyme within the glutathione peroxidase family, playing a key role in various biological processes. It primarily converts lipid peroxides into lipid alcohols, thereby protecting cells from oxidative stress damage. GPX4 is overexpressed in various cancers, including non-small cell lung cancer (NSCLC), and is associated with tumor invasion, metastasis, and drug resistance. In 2023, Hu et al. reported the first GPX4-based fluorescent probe ENBO-ML210 (Figure 4) for the imaging of NSCLC [43]. Upon entering the body, the NIR fluorescence group Nile blue (NB) in the probe binds to the cell membrane through electrostatic interactions, facilitating rapid cellular uptake. The GPX4 inhibitor ML210yne specifically binds to GPX4, resulting in the enrichment of the probe in tumor cells and a higher fluorescence intensity in tumor cells compared to normal cells. This differential fluorescence intensity enables the specific imaging of tumor cells.

**Figure 5:**
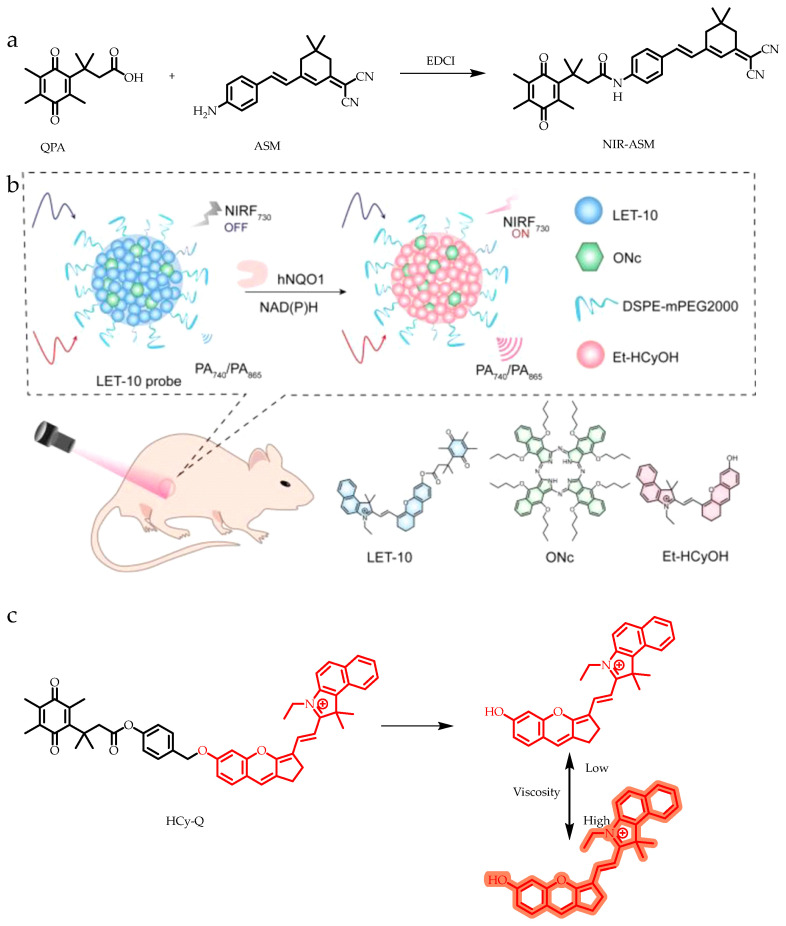
(**a**) OPA and ASM form IR-ASM in EDCI; (**b**) Structure of LET-10 and the fluorescence activation process under the influence of hNQO1, reproduced from [45] with permission from American Chemical Society, Copyright 2022; (**c**) Structure of HCy-Q and the fluorescence activation process under the influence of NQO1 in the presence of NADH. EDCI: 1-(3-Dimethylaminopropyl)-3-ethylcarbodiimide hydrochloride; hNQO1: human quinone NADH dehydrogenase 1; PA: photoacoustic; DSPE-mPEG2000: a liposome material composed of amphiphilic phosphatidylethanolamine (DSPE) and hydrophilic polyethylene glycol (PEG2000) was used as a coating material to wrap around the LET-10 probe to form nanoparticles.

Mitochondria serve as the energy centers of cells, providing capacity through respiration. Quinone NADH dehydrogenase 1 (NQO1) plays a crucial role in this process. High expression of NQO1 is closely associated with the development of various tumors. In 2019, Punganuru et al. reported that the fluorescent probe NIR-ASM (Figure 5a) that responds to NQO1 successfully imaged an A549 tumor model mice in vivo [44]. The team conducted in vivo imaging of A549 and LLC (a mouse lung cancer cell line) tumor model mice by detecting endogenous NQO1 levels. They constructed the complete probe by coupling the NQO1 substrate quinone propionic acid (QPA) with the fluorophore dicyanoisophorone (ASM). The probe exhibited high specificity, sensitivity, and good biocompatibility in both cell and animal models. In 2022, Zhang et al. reported LET-10 (Figure 5b), an NIR fluorescence and ratiometric photoacoustic dual-mode imaging probe based on hemi-cyanine dye, for the early diagnosis and prognosis assessment of lung cancer [45]. The quinone propionic moiety in the probe is subject to hNQO1-specific enzyme activity, which enhances the NIR fluorescence and photoacoustic (PA) signals. Octabutoxy naphthalocyanine (ONc) in the probe serves as an internal parameter for photoacoustic imaging, maintaining a constant photoacoustic signal and enabling ratiometric photoacoustic imaging. The successful application of LET-10 in an hNQO1-positive A549 tumor model demonstrated its ability to achieve high sensitivity and high contrast detection of hNQO1 activity, making it a promising tool for early diagnosis and prognosis assessment of lung cancer. Similarly, Cao et al. reported the fluorescent probe HCy-Q (Figure 5c) in 2024, which can target mitochondria and facilitate the in vivo imaging of A549 tumor models by responding to NQO1 [46]. The probe comprises a semi-cyanine dye, HCy, and an NQO1 recognition group. The initial fluorescence of HCy is suppressed by the photoinduced electron transfer (PET) process. Additionally, due to its unique D-π-A structure, the probe is sensitive to the viscosity of the mitochondrial membrane and can reflect the cellular apoptosis process by monitoring viscosity changes. The sensitivity of the D-π-A structure to the viscosity of the mitochondrial membrane can be explained by the ICT mechanism. When the viscosity of the mitochondrial membrane varies, the degree of intramolecular rotation of the probe molecule also changes, resulting in alterations to the probe’s fluorescence intensity. Consequently, the probe can simultaneously perform specific imaging of tumor cells and monitor the apoptotic process, which is significant for studying the role of mitochondria in tumor development and diagnosis.

**Figure 6:**
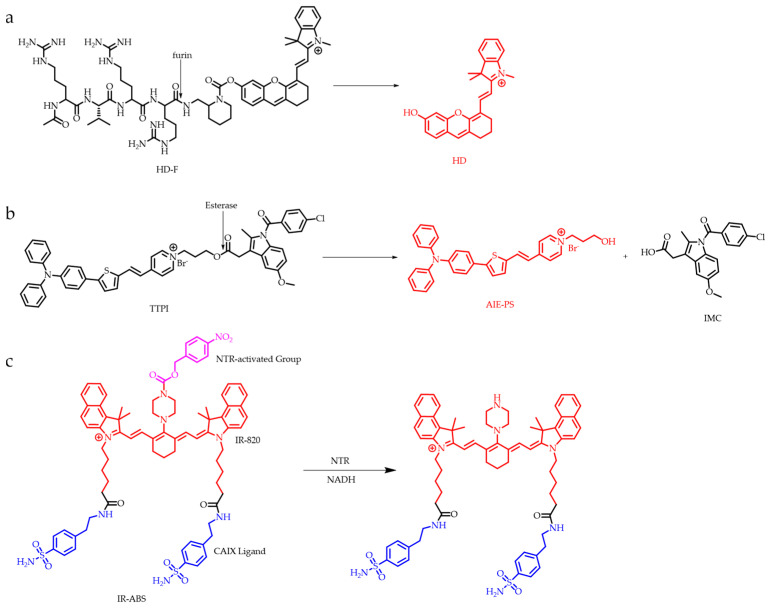
(**a**) Structure of HD-F and the fluorescence activation process influenced by furin; (**b**) Structure of TTPI and schematic of IMC and AIE-PS release process influenced by esterase; (**c**) Structure of IR-ABS and the schematic of the fluorescence activation process after the active targeting of tumor cells mediated by CAIX under the combined action of NTR and NADH. NTR: nitroreductase.

The Golgi apparatus is a vital organelle involved in the processing, modification, sorting, and transportation of proteins, serving as a key hub for intracellular material transport and signal transmission. Additionally, it is involved in the proliferation, invasion, and metastasis of tumor cells. Furin is a critical protease predominantly located in the Golgi apparatus. It belongs to the protein invertase family and performs various biological functions in cells, particularly in tumorigenesis and development. Furin expression is typically higher in ovarian cancer, breast cancer, and NSCLC cells compared to normal cells. To investigate furin’s role in carcinogenesis and tumor imaging, Zhu et al. reported a fluorescent probe, HD-F (Figure 6a), that targets the Golgi apparatus in 2019 [47]. Upon specific recognition and enzymatic cleavage of the polypeptide sequence RVRR by furin, NIR fluorophore HD is released, activating the fluorescence signal. Furin detection and imaging can then be achieved by measuring the fluorescence signal at 708 nm, accurately indicating the tumor’s location. In 2024, Wang et al. reported a multifunctional fluorescent probe, TTPI (Figure 6b), capable of targeting the Golgi apparatus [48]. TTPI integrates multiple functions, including NIR fluorescence imaging, photodynamic therapy, and inhibition of cyclooxygenase-2 (COX-2)—an inducible enzyme overexpressed in many cancers that promotes tumor cell proliferation, invasion, and metastasis and inhibits apoptosis. Additionally, as COX-2 expression also increases during inflammation, the researchers employed the reported IMC-conjugated fluorescence assay to differentiate between inflammation and tumors during probe construction. Upon targeting and aggregating in the Golgi apparatus, TTPI produces a strong fluorescence signal due to aggregation-induced emission (AIE), thus enabling the real-time imaging of cells. Simultaneously, light excitation generates a substantial amount of reactive oxygen in situ, effectively killing tumor cells. Indomethacin (IMC), a COX-2 inhibitor included in the probe, also inhibits COX-2 activity and reduces tumor metastasis, thereby achieving selective imaging and efficient eradication of cancer cells. The researchers thoroughly validated the performance of TTPI through experiments involving A549 tumor animal models, thereby providing robust support for subsequent clinical trials.

Compared to traditional intravenous or oral administration, tracheal inhalation delivers drugs directly to the lungs for the diagnosis and treatment of lung cancer, increasing drug concentration in the lungs while reducing systemic exposure. This method also allows drugs to enter the blood circulation directly, improving bioavailability. Additionally, it is easy to operate and suitable for various stages of lung cancer, from early to late stages. The hypoxic characteristics of the tumor microenvironment (TME) are closely associated with tumor progression, with nitroreductase (NTR) and carbonic anhydrase IX (CAIX) being highly expressed under hypoxic conditions. These enzymes synergistically promote tumor cell proliferation and metastasis, making them potential targets for tumor diagnosis. Inspired by these findings, Yan et al. developed a novel inhaled NIR fluorescent probe, IR-ABS (Figure 6c), for NSCLC diagnosis in 2023 [49]. In the probe, chloroformic acid-4-nitrobenzyl ester is reduced by NTR to release IR-820 fluorophores, dissolving the PET process and activating the fluorescence signal. Additionally, 4-(2-aminoethyl) benzenesulfonamide specifically targets CAIX. The probe accurately distinguishes tumor tissue from normal tissue in various tumor models, including in situ lung cancer animal models, and clearly identifies hypoxic regions of tumor tissue. This tracheal inhalation method provides a new direction and perspective for the diagnosis and treatment of lung cancer.

This section summarizes some common fluorescent probes associated with proteases in lung cancer. These probes are characterized by their enzyme responsiveness. When these probes accumulate in the tumor and react with enzymatic biomarkers, their fluorescence is activated, classifying them as activatable fluorescent probes. (i) Highly specific, activatable fluorescent probes must interact with tumor-associated enzymatic biomarkers before activation and fluorescence. This interaction significantly reduces background signal and enhances the signal-to-noise ratio, thereby improving tumor detection accuracy. (ii) Low background signal: In their inactive state, these probes do not fluoresce, which reduces background noise and enhances imaging clarity. This feature is particularly important for in vivo imaging, as it minimizes interference from non-specific fluorescence signals. (iii) Real-time imaging capabilities: Activatable fluorescent probes enable real-time monitoring of tumor dynamics in vivo, providing researchers and clinicians with more accurate insights into tumor progression and treatment effects. (iv) Multi-probe strategy: A range of activatable fluorescent probes can be designed to target different tumor-associated enzymes, enabling multidirectional imaging of lung cancer or lung cancer cells. However, these probes also have limitations; for instance, the targeted enzymes are not exclusively expressed in tumor cells. Furthermore, low expression levels of these enzymes may challenge the probe’s sensitivity. Additionally, some fluorescent probes may exhibit limited biocompatibility in vivo, potentially triggering immune responses or other adverse reactions. These challenges complicate the design and synthesis of these probes. It is essential to develop strategies to mitigate misdiagnosis or false positives and to enhance the biocompatibility of these probes.

### 3.2. Receptor-Binding Fluorescent Probes

**Figure 7:**
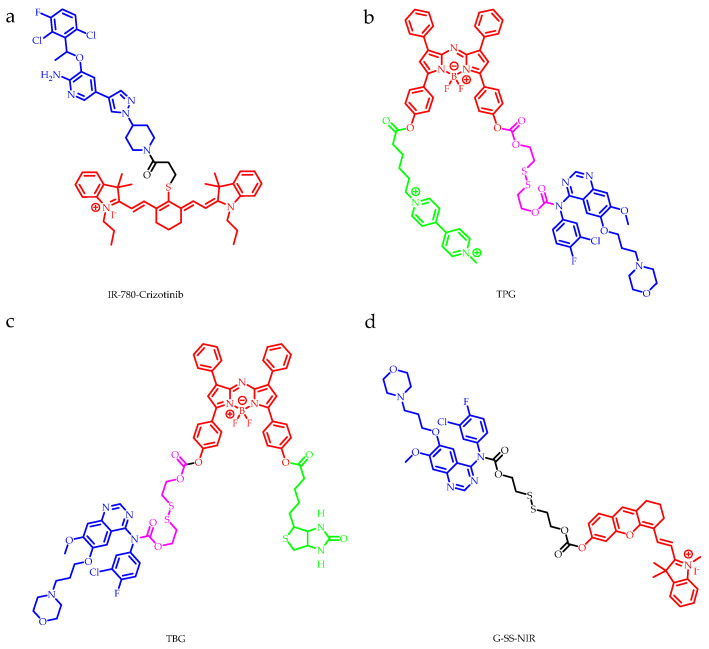
(**a**) Structure of IR-780-Crizotinib; (**b**) Structure of TPG; (**c**) Structure of TBG; (**d**) Structure of G-SS-NIR. Blue: chemotherapy drugs; Green: targeting ligand; Purple: linker; Red: NIR fluorophores.

Anaplastic lymphoma kinase (ALK) is a transmembrane receptor tyrosine kinase that is highly expressed in various tumor cells. ALK supports tumor development by promoting cell proliferation, differentiation, survival, inhibiting apoptosis, enhancing cell invasion and metastasis, and stimulating angiogenesis. Therefore, ALK represents a potential therapeutic target [50,51,52]. In 2021, Wang et al. developed a multifunctional fluorescent probe, IR-780-Crizotinib (Figure 7a), by covalently linking the NIR dye IR-780 with the chemotherapeutic drug Crizotinib [53]. This probe combines imaging and therapeutic functions. The team utilized Crizotinib’s ability to target ALK to localize and concentrate IR-780 within NSCLC cells. The presence of IR-780 in the probe enabled NIR fluorescence emission, while Crizotinib’s specific binding to ALK inhibited relevant signaling pathways, resulting in reduced tumor cell growth and proliferation. Both cell and animal model experiments demonstrated that the probe exhibits favorable in vivo metabolism and shows promising application potential.

Epidermal growth factor receptor (EGFR), a member of the ErbB receptor tyrosine kinase family, is implicated in various malignant tumors. Abnormal EGFR expression is found in over 70% of malignant tumors, such as bladder, lung, and prostate cancers, making it an ideal target for tumor diagnosis and treatment. Glutathione (GSH) is a reactive sulfur species (RSS) commonly found in organisms, and its elevated expression is associated with various tumors, including lung cancer [54,55]. Combining GSH and EGFR to develop fluorescent probes is a significant undertaking. Therefore, in 2018, Song et al. reported that a fluorescent probe, TPG (Figure 7b), can induce the apoptosis of NSCLC cells by inhibiting the EGFR signaling pathway. The probe consists of a targeted prodrug, PPG (integrated Gefitinib, a chemotherapy drug), targeting ligand polyamine analog (PA), and the NIR fluorophore azo-BODIPY, connected by a disulfide bond to form a complete probe. In vivo, PA targets tumor cells and is taken into cells. The high levels of GSH and disulfide bonds in tumor cells release the Gefitinib and azo-BODIPY components, enabling the in vivo imaging of tumors through fluorescence signals and real-time monitoring of drug release. TPG’s therapeutic efficacy was improved, and it showed cytotoxicity in cell lines resistant to tyrosine kinase inhibitors (TKI). In 2020, Xie et al. reported G-SS-NIR (Figure 7d), a fluorescent probe similar to TPG, which also contained Gefitinib [56]. Additionally, the probe can self-assemble into nanoparticles in water. The nanoparticle drug CEL@G-SS-NIR was formed by coating Celastrol, another chemotherapeutic drug with functions similar to Gefitinib. Gefitinib and Celastrol inhibit the upstream and downstream EGFR signaling pathways, respectively, which is beneficial for improving the treatment of TKI-resistant NSCLC. In 2022, Song’s team expanded on TPG’s work to develop an NIR fluorescent probe called TBG (Figure 7c), utilizing a strategy similar to TPG [57]. TBG and TPG both exhibit effective targeting and therapeutic effects, and can monitor drug delivery and treatment processes in real-time. These three different probes suggest novel strategies and directions for the treatment and prognosis of NSCLC.

**Figure 8:**
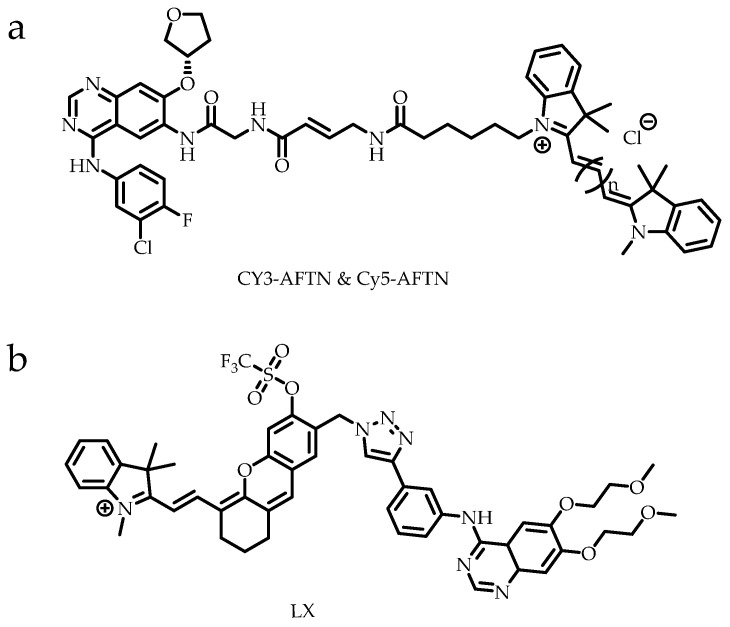
(**a**) Structure of CY3-AFTN and Cy5-AFTN; (**b**) Structure of LX.

HER1/HER2 are crucial members of the EGFR family. In 2018, Liu et al. reported the development of two fluorescent probes, CY3-AFTN and Cy5-AFTN (Figure 8a), based on the clinical drug afatinib [58]. These probes demonstrated effective aggregation and imaging in A549 nude mouse tumor models. The team introduced amino acid residues to afatinib to achieve reversible binding of the probe to HER1/HER2. The presence of a long flexible side chain between afatinib and the Cy dye ensures that the fluorescence characteristics of the Cy dye remain unaffected by binding to HER1/HER2. This unique design allows the probe to be competitively replaced by other HER1/HER2 inhibitors while maintaining excellent fluorescence properties. Consequently, the probe can be used not only for imaging but also for high-throughput drug screening.

Simultaneously, EGFR tyrosine kinase inhibitors (EGFR-TKI) are an effective treatment method for NSCLC; however, this method has some limitations that cannot be ignored. For example, due to individual genetic differences and drug resistance, only about 20% of patients show improvement after receiving EGFR-TKI treatment. Additionally, EGFR-TKI resistance in tumor cells is closely related to reactive oxygen species (ROS). Therefore, in 2023, Lu et al. reported a hemicyanine derivative-based fluorescent probe, LX (Figure 8b), for EGFR-TKI suitability screening and resistance monitoring [59]. The team used EGFR-TKI (erlotinib) as the targeted component of the probe, while the triflate in the probe acts as the ROS-responsive part and also quenches the initial fluorescence of the probe through the PET process. Due to the probe’s EGFR targeting and correlation with ROS concentration, it is possible to monitor EGFR-TKI suitability and drug resistance in cells through fluorescent signals. If ROS levels in tumor cells are high, the probe will fluoresce, indicating that the tumor cells are sensitive to EGFR-TKI and that the patient is suitable for EGFR-TKI therapy. If ROS levels in tumor cells are low, the probe will not fluoresce, indicating that the tumor cells are not sensitive to EGFR-TKI and that the patient is not suitable for EGFR-TKI treatment. This can screen patients for EGFR-TKI suitability and detect EGFR-TKI resistance in a timely manner, guiding the adjustment of clinical treatment plans to seek the optimal treatment.

This section summarizes receptor-related fluorescent probes. Compared with enzyme-activated fluorescent probes, the ligands specific to receptors in these probes enhance targeting, significantly reducing the probability of misdiagnosis due to the high expression of these receptors in tumor cells. While these probes offer clear advantages, they also have notable disadvantages: (i) these receptors are expressed in non-tumor cells as well; (ii) these receptors may bind to their endogenous ligands before interacting with the probe, which can diminish the probe’s targeting efficiency; (iii) the stability and in vivo biocompatibility of the probes require careful consideration. However, these probes present additional advantages over enzymatic probes; notably, many of these receptor-targeted probes utilize inhibitors that inherently possess tumor-suppressive and cytotoxic properties, giving most of the probes discussed in this section therapeutic potential. Thus, these probes hold significant potential as references for the future development of multifunctional probes that combine therapeutic and imaging capabilities.

### 3.3. Antigen-Based Probes

Carcinoembryonic antigen (CEA) is a glycoprotein, and the serum CEA concentration in patients with colon cancer, breast cancer, and lung cancer is significantly higher than that in healthy individuals. Sensitive detection of CEA in serum is crucial for clinical screening and the early diagnosis of cancer. However, many existing methods have limitations, such as poor stability, time-consuming procedures, and inaccurate localization. Additionally, CYFRA21-1, a fragment of cytokeratin 19 (CK19), is highly expressed in epithelial tumors such as NSCLC, leading to elevated CYFRA21-1 levels in the serum of patients with NSCLC. Thus, CYFRA21-1 serves as an important biomarker for lung cancer. Monitoring the serum levels of CYFRA21-1 in patients can be employed for the diagnosis and monitoring of NSCLC, as well as for evaluating the efficacy of treatments [60,61,62].

**Figure 9:**
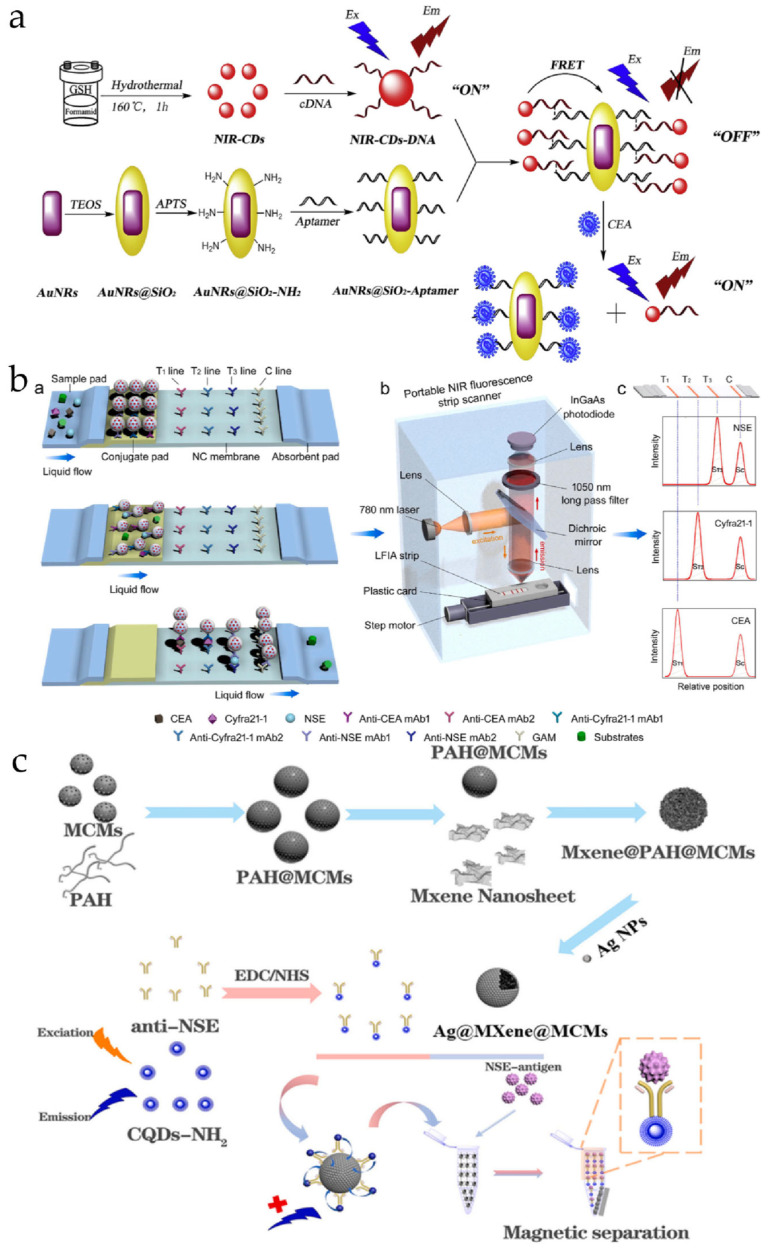
(**a**) The construction process and principle of the NIR fluorescence probe by Shao’s team, reproduced from [63] with permission from Elsevier, Copyright 2019; (**b**) LFIA platform construction diagram of Li’s team, reproduced from [65] with permission from Elsevier, Copyright 2022; (**c**) Schematic of the construction process of anti-NSE/CQDs/Ag@MXene@MCMs (the construction process of anti-CEA/QDs/Ag@MXene@MCMs is similar), reproduced from [66] with permission from Elsevier, Copyright 2024. In subfigure (**b**), a: Principle of using NFC-LFIA test strips to detect multi-indicator lung cancer biomarkers. b: Portable NIR-II fluorescence scanner supporting NFC-LFIA test strips. c: Representative scanning curves of fluorescence signals on the test line and control line of the LFIA test strip.

In 2019, Shao et al. developed a fluorescence probe, NIR-CDs-DNA-AuNRs@SiO_2_-Aptamer (Figure 9a), that responds to CEA using the fluorescence resonance energy transfer (FRET) principle [63]. The team first synthesized AuNRs@SiO_2_-Aptamer and NIR-CDS-DNA, which were joined by a preformed double-stranded DNA structure to build the complete probe. In the presence of CEA, the aptamer in AuNRs@SiO_2_-Aptamer specifically binds to CEA, resulting in the unspinning of the previous DNA double-strand, which separates NIR-CDS-DNA from the surface of AuNRs@SiO_2_. Consequently, the quenching effect of AuNRs@SiO_2_-Aptamer disappears, activating fluorescence. In the detection of CEA in pleural effusion, the probe demonstrated a linear correlation with CEA concentration, high sensitivity (detection limit as low as 0.02 pg/mL), and high specificity, while also exhibiting good repeatability. This provided a useful tool and direction for the early diagnosis of lung cancer by detecting CEA concentration in pleural effusion.

In 2023, Hou et al. reported an NIR biosensor platform (labeled as Probe 19 in this article) for ultra-sensitive linear monitoring of CYFRA21-1, with a detection limit of 38.7 fg/mL [64]. The team utilized upconversion nanomaterials (UCNPs), which possess NIR luminescence properties, as the core of the platform. They combined this with atom transfer radical polymerization (ATRP), a controllable/active radical polymerization technology, to realize the complete function of the platform. The detection process can be divided into four steps: (1) The sample is combined with MNPs-Ab1 through pretreatment to form Ab1-CYFRA21-1 Complex 1; (2) Complex 1 is mixed with Ab2/BIBA combined with an ATRP initiator to obtain Ab1-CYFRA21-1-Ab2/BIBA Complex 2; (3) A large number of UCNPs/HEMA are assembled on Ab2/BIBA through ATRP reaction to form MNPs-Ab1-CYFRA21-1-Ab2/BIBA-HEMA/UCNPs Complex 3; (4) Finally, the fluorescence intensity of Complex 3 is detected to determine the concentration of CYFRA21-1, thereby achieving the detection and evaluation of NSCLC.

Lateral Flow Immunoassay (LFIA) is an in vitro diagnostic technique based on antigen–antibody immune responses. In addition to CEA and CYFRA21-1, NSE is another important biomarker of lung cancer, involved in the glycolysis process and playing a crucial role in cellular energy metabolism. Elevated levels of NSE are associated with certain types of lung cancer, particularly SCLC. In 2022, Ao et al. combined NIR fluorescence imaging technology with LFIA to build a platform (Figure 9b) (labeled as Probe 20 in this article) that can simultaneously detect CEA, CYFRA21-1, and NSE for lung cancer diagnosis [65]. The team first synthesized a fluorescent labeling material based on lead sulfide quantum dots (PbS QDs) and used it as a platform for an NIR II fluorescence probe (NFC). They modified amino and carboxyl functional groups on the surface of NFC to enable conjugation with antibodies. Then, the cellulose nitrate (NC) membrane was coated with trapping antibodies for CEA, CYFRA21-1, and NSE binding to NFC. Finally, all the components were assembled together to form the LFIA test strip. The platform demonstrated high sensitivity, high specificity, and good reproducibility in subsequent performance evaluation experiments, accurately and quickly detecting lung cancer biomarkers and distinguishing lung cancer patients from healthy individuals, with sensitivity and specificity reaching 92.7% and 92.0%, respectively. This method provides a fast and convenient approach for lung cancer diagnosis.

In 2024, Yin et al. designed a probe base vector (Figure 9c) (labeled as Probe 21 in this article) to achieve the specific detection of CEA or NSE by incorporating different probes [66]. The team first used a novel type of MXene material, a two-dimensional material composed of metallic carbides or nitrides, similar to graphene in its layered structure. Specifically, Ti3C2Tx was bonded with pre-prepared magnetic carboxyl microspheres (MCMs) using poly (allylamine hydrochloride) (PAH) to form MXene@MCMs. Subsequently, the basic carrier Ag@MXene@MCMs was obtained by leveraging the reducibility of MXene to support silver nanoparticles (AgNPs) on the surface of MXene@MCMs. The probe was partially constructed by associating amino carbon quantum dots (CQDs) with antibodies corresponding to NSE and CEA. Finally, the probes corresponding to NSE and CEA were combined with AgNPs on the base carrier to obtain anti-NSE/CQDs/Ag@MXene@MCMs or anti-CEA/CQDs/Ag@MXene@MCMs. When the corresponding biomarker is present and forms an antigen–antibody complex, CQDs will detach from the surface of AgNPs, resulting in fluorescence recovery. This “modular” design allows the probe to be tailored to different application environments, offering significant reference value for the future development of NIR fluorescence imaging technology.

Some of the antigen-related sensors discussed in this section are designed for in vitro use, which presents a clear disadvantage: they cannot monitor tumor presence and dynamics in vivo. Additionally, antigens commonly take the form of proteins, polysaccharides, glycoproteins, or nucleic acids, making it crucial to effectively screen and select appropriate antigens from among numerous biomolecules for use as detection targets. For example, CYFRA21-1, discussed in this section, is a specific antigen predominantly expressed in NSCLC. In contrast to CEA, which is expressed in various tumor types, CYFRA21-1 is more targeted, yielding more reliable results. Furthermore, in vitro detection allows for less restrictive sensor design, as researchers need only focus on accurately and sensitively detecting the relevant biomarkers without the complications associated with in vivo applications. Although these sensors have clear disadvantages, the convenience they offer during probe construction, particularly in avoiding the complexities of in vivo design, is unmatched by other types of internal probes.

### 3.4. Probes Based on Cell Physical Properties

pH, viscosity, and polarity exhibit significant differences between tumor cells and normal cells. These parameters can serve as crucial diagnostic indicators for tumors. Leveraging these differences as diagnostic markers provides a feasible approach for distinguishing tumor cells from normal cells, thereby facilitating the diagnosis of lung cancer [67,68,69,70].

**Figure 10:**
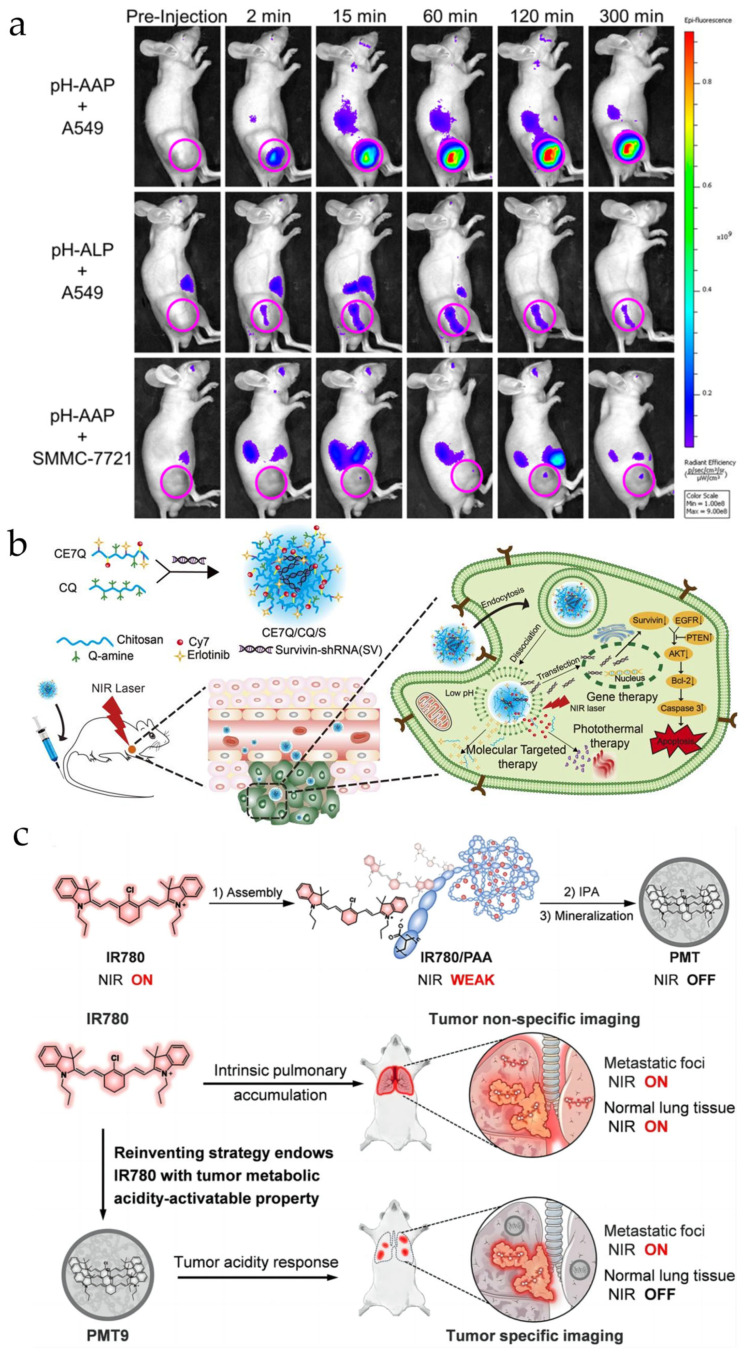
(**a**) Schematic of pH-AAP imaging results in different tumor mouse models, reproduced from [71] with permission from American Chemical Society, Copyright 2019; (**b**) Construction process and mechanism of CE7Q/CQ/S, reproduced from [72] with permission from Elsevier, Copyright 2020; (**c**) The construction process of PMT and the advantages of the mechanism, reproduced from [73] with permission from John Wiley and Sons, Copyright 2023.

In 2019, Shi et al. successfully imaged an animal model of A549 using pH-AAP (Figure 10a), a pH-responsive bi-specific fluorescent probe [71]. The team first selected aptamers—short nucleic acid molecules capable of recognizing cell surface receptors, proteins, and small molecules—from a pool of random sequences using in vitro screening (SELEX) technology. The selected aptamer was modified with Cy5, an NIR fluorescent dye, to form COOH-Cy5-DNA, which was then combined with an acid-labile acetal linker serving as the pH response site. This led to the formation of ATU-Cy5-DNA. Finally, by linking ATU-Cy5-DNA with NHS-BHQ, they constructed the complete fluorescence probe BHQ2-ATU-Cy5-DNA (pH-AAP). BHQ efficiently quenched the initial fluorescence of Cy5, providing the probe with a very low background signal. The probe can be activated through two mechanisms. First, upon injection into the body, the aptamers specifically recognize and accumulate on the receptors of tumor cells, directly responding to the acidic tumor microenvironment. Second, the probe is absorbed into the tumor cells and further activated within the acidic lysosome, resulting in enhanced fluorescence. Both in vitro and in vivo experiments demonstrated that pH-AAP has an ultra-low background signal and excellent fluorescence stability, enabling non-wash, bi-specific, and contrast-enhanced tumor imaging. This promising molecular probe is expected to be utilized for early diagnosis and treatment monitoring. Functional integration is a major trend in the development of fluorescent probes. In 2020, Li et al. developed CE7Q/CQ/S (Figure 10b), a multifunctional nanoparticle that is pH-sensitive within the tumor microenvironment [72]. This nanoparticle integrates chemotherapy, gene therapy, photothermal therapy, and NIR fluorescence imaging. The team first combined erlotinib (Er), a chemotherapy drug, with heptamethine cyanine dye (Cy7), an NIR photothermal converter capable of converting absorbed NIR light into heat to kill tumor cells, and quaternary ammoniums, conjugated to chitosan (CS) to form CE7Q. CE7Q was then combined with 6-N,N,N-trimethyltriazole chitosan (CQ), which has an excellent gene transfection efficiency, and a plasmid expressing survivin shRNA (SV). Survivin is an apoptosis suppressor protein whose overexpression is associated with the progression and drug resistance of NSCLC; survivin shRNA can specifically silence the survivin gene to promote the apoptosis of tumor cells and enhance the efficacy of chemotherapeutic drugs. The complete CE7Q/CQ/S fusion enables triple therapy mediated by Er, SV, and Cy7. Upon entering NSCLC cells, CE7Q/CQ/S releases the encapsulated drug in a low-pH environment under NIR light irradiation. This process silences the survivin gene, induces the photothermal effect of Cy7, and delivers the targeted chemotherapy effect of Er, effectively inhibiting EGFR-mutated NSCLC cells and achieving high efficacy. These results suggest that the chemotherapeutic/gene/photothermal triple therapy of CE7Q/CQ/S may provide an effective strategy for overcoming intrinsic or acquired EGFR-TKI resistance in NSCLC.

At the same time, lung metastasis is one of the primary causes of death among lung cancer patients, and the slightly acidic characteristics of the tumor microenvironment (TEM) can be utilized to develop tumor imaging probes with enhanced specificity. In 2023, Jia et al. developed an NIR fluorescence probe, PMT9 (Figure 10c), based on the NIR dye IR780 and calcium phosphate (CaP) [73]. The probe specifically accumulates in tumor tissue through blood circulation. Meanwhile, CaP nanoparticles dissociate at pH 6.8–7.0 to release IR780 and activate NIR fluorescence, thereby achieving the non-active specificity of PMT9. PMT9 can effectively distinguish tumor tissue from normal tissue in subcutaneous transplantation lung cancer models, in situ lung cancer models, and breast cancer lung metastasis models. This indicates that PMT9 can accurately detect lung metastatic tumors, providing a novel approach for the diagnosis of lung cancer and the application of image-guided therapy.

**Figure 11:**
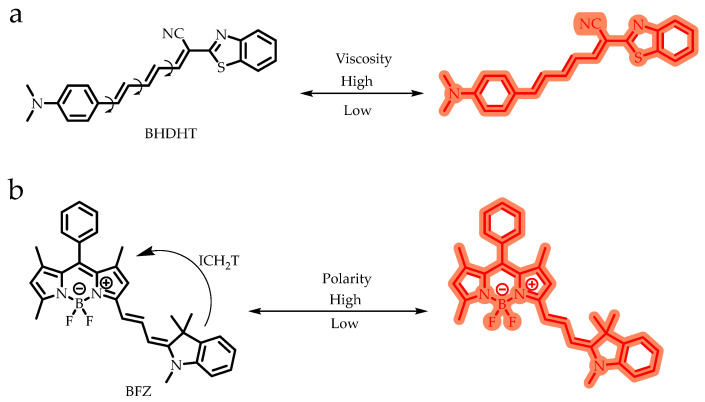
(**a**) Structure of BDHT; (**b**) Structure of BFZ.

Lipid droplets are important organelles in the cell, primarily responsible for storing neutral lipids (such as triglycerides, cholesterol esters, and fatty acids). Recent studies have shown that they are closely related to ferroptosis. However, the changes in lipid droplet viscosity during ferroptosis remain unclear. In 2020, Dong et al. reported a fluorescent probe, BDHT (Figure 11a), that can display changes in lipid droplet viscosity during ferroptosis in various tumor cells, including A549 [74]. The probe enters the cell via passive diffusion and primarily accumulates in lipid droplets. Due to the unique conjugation system of BDHT molecules, intramolecular rotation occurs, affecting the fluorescence intensity. Specifically, when the viscosity of the lipid droplets increases, the intramolecular rotation is restricted, resulting in enhanced fluorescence intensity. When the viscosity of the lipid droplets decreases, the intramolecular rotation becomes freer, resulting in a decrease in fluorescence intensity. By detecting changes in fluorescence intensity, the process of lipid droplet viscosity changes can be monitored in real time and in situ. BDHT is highly sensitive to viscosity changes and can detect minute changes, providing a powerful tool for studying lipid droplet function and the mechanisms of ferroptosis in tumor cells.

Changes in lipid droplet polarity are also closely related to various diseases (such as tumors and inflammation), but the mechanisms remain unclear. Therefore, developing an effective method to detect lipid droplet polarity is crucial for studying the progression of pneumonia and lung cancer. In 2024, Zhang et al. reported BFZ (Figure 11b), a fluorescent probe designed to target lipid droplets [75]. BFZ can monitor changes in lipid droplet polarity and differentiate between tumor and normal cells based on this polarity. The probe’s unique D-π-A molecular structure allows it to respond to changes in polarity through intramolecular charge transfer (ICT) properties. In this structure, Fischer aldehyde serves as the electron donor (D), while fluoroborondipyrrole (BODIPY) functions as the electron acceptor (A). The complete BFZ probe is formed by connecting the two components through a π-conjugated bridge. In both cell and animal model experiments, BFZ successfully differentiated tumor cells from normal cells, with significantly higher fluorescence intensity observed in tumor tissue compared to normal tissue. The successful application of BFZ offers new insights and directions for studying the progression of pneumonia and lung cancer.

Physicochemical properties have been a major focus in tumor-related studies, with fluorescent probes developed to target these parameters aiding in the elucidation of mechanisms underlying tumor progression. Compared with the three types of fluorescent probes previously discussed, these probes have relatively general targeting capabilities. However, they are highly sensitive to subtle changes in physicochemical parameters and are not influenced by the expression levels of other substances, partially compensating for their limited targeting specificity. Additionally, the design of these probes is complex and requires optimization in terms of excitation wavelength, fluorescence lifetime, and photostability to adapt to the intricate in vivo environment. Moreover, these probes have a limited response range and may not fully capture all environmental changes. Clinical translation presents additional challenges. Despite their strong laboratory performance, these probes can be influenced by factors such as inflammation and metabolic changes, leading to increased background signals and reduced imaging accuracy. Therefore, in clinical applications, the complex in vivo microenvironment and individual patient differences may affect the probes’ effectiveness and reliability, complicating their clinical translation.

### 3.5. Other Fluorescent Probes Related to Lung Cancer

In addition to the aforementioned probes, several other distinctive fluorescent probes have been reported for lung cancer. Compared with the four types of probes previously discussed, these probes offer additional advantages, such as responding to active substances within tumors (e.g., reactive oxygen species, amino acids) or achieving functional integration with gene therapy through innovative design. These distinctive fluorescent probes are summarized in this section.

**Figure 12:**
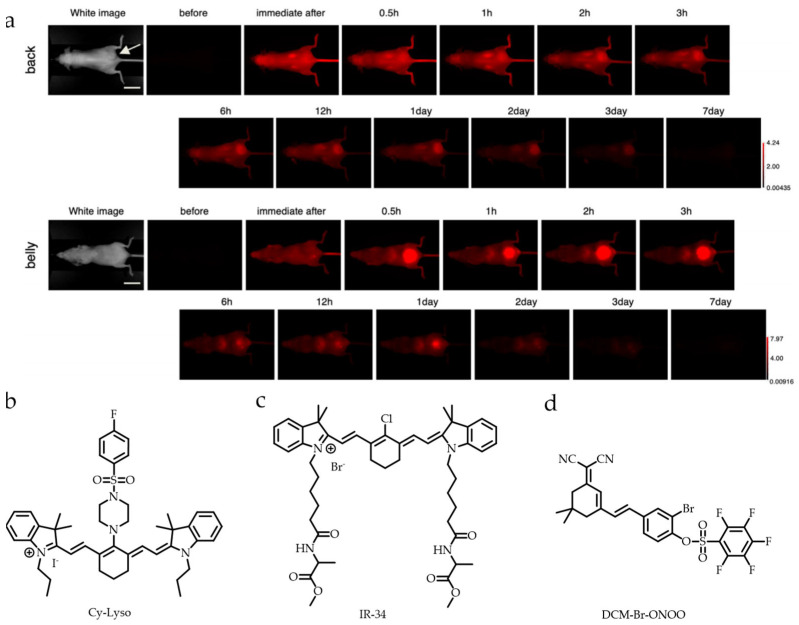
(**a**) Schematic of the distribution of rova-IR700 in the body and its metabolic time after intravenous administration, reproduced from [76] with permission from Elsevier, Copyright 2020; (**b**) Structure of Cy-Lyso; (**c**) Structure of IR-34; (**d**) Structure of DCM-Br-ONOO.

DLL3 (Delta-like protein 3) is a protein that is highly expressed in SCLC but is minimally expressed in other cancer types and normal tissues. It acts as a ligand for the NOTCH signaling pathway and activates this pathway by binding to the NOTCH receptor. The NOTCH signaling pathway plays a critical role in the proliferation, differentiation, and survival of tumor cells. Consequently, DLL3 is crucial in the occurrence and progression of SCLC, potentially influencing tumor cell proliferation, invasion, and metastasis. In 2020, Isobe et al. paired the NIR photosensitizer IR700 with the anti-DLL3 monoclonal antibody Rovalzumab to create rova-IR700 (Figure 12a) for targeted NIR photo immunotherapy (NIR-PIT) of SCLC [76]. This probe significantly inhibits tumor growth and prolongs survival in SCLC xenografted models in nude mice. Additionally, Furumoto et al. summarized the potential applications of NIR-PIT for treating thoracic, abdominal, and respiratory cancers in 2022 [77]. They highlighted that NIR-PIT offers higher specificity compared to traditional photodynamic therapy and avoids damage to surrounding normal tissues while specifically targeting cancer cells. Importantly, NIR-PIT induces immunogenic cell death (ICD) and activates the host’s anti-tumor immune response. The integration of immunotherapy with NIR fluorescence probes provides valuable insights for developing efficient and convenient treatment strategies in the future.

Lysosomal dysfunction may be linked to the occurrence and progression of certain cancers, including lung and gastric cancer. In 2020, Li et al. reported the NIR fluorescent probe Cy-Lyso (Figure 12b) designed for visualizing and tracking the metastasis of lung cancer cells [78]. The probe comprises an NIR fluorescent group (Cy) and a p-fluorobenzenesulfonyl group connected via piperazine. The p-fluorobenzenesulfonyl group in the probe specifically binds to a receptor on the lysosomal membrane. Concurrently, the piperazine moiety within the probe can bind to a significant amount of H⁺ in the lysosome, thereby further enhancing the probe’s targeting capability. The synergistic effects of these factors enable the probe to actively target the lysosome. Furthermore, as the fluorescent group emits an NIR signal, the lysosome can be visualized by NIR fluorescence imaging. Simultaneously, the strong binding of the p-fluorobenzenesulfonyl group to the lysosomal membrane endows the probe with prolonged imaging capabilities, as well as the ability to track dynamic changes in living cells, such as cell migration and division. Consequently, the probe can monitor the metastatic processes of tumor cells and provide more detailed insights into the mechanisms of tumor metastasis. In in vivo experiments, the team observed that probe-labeled A549 cells metastasized to the liver and intestine, indicating that the probe serves as an effective tool for both basic and clinical research on single-cell behavior. This advancement aids in understanding the mechanisms of lung cancer cell metastasis and offers new avenues for cancer treatment.

The endoplasmic reticulum (ER) is a crucial organelle involved in various cellular functions, including cell signaling, apoptosis, phospholipid and cholesterol synthesis, drug metabolism and detoxification, and protein processing and transport. Endoplasmic reticulum stress (ERS) is commonly observed in tumor cells and is closely associated with the onset and progression of numerous diseases. ERS can facilitate tumor development to some extent; however, prolonged and excessive ERS may directly induce tumor cell death [79,80,81,82]. In 2018, Wang et al. reported that the NIR fluorescent dye IR-34 (Figure 12c) not only facilitates NSCLC imaging but also targets the mitochondrial protein NDUFS1, a subunit of mitochondrial complex I in tumor cells [83]. Mitochondrial complex I, a component of the mitochondrial electron transport chain, transfers electrons from NADH to ubiquinone, creating a proton gradient that generates energy for ATP synthesis. Additionally, the interaction between heptamethine and NDUFS1 in IR-34 can impair mitochondrial function and enhance the production of reactive oxygen species (ROS). The combined effects of ERS and ROS can effectively induce tumor cell death and prevent recurrence. However, the mechanisms underlying ERS and tumor development are not fully understood, with ONOO^−^ being a significant factor contributing to ERS. To further investigate this mechanism, In 2024, Yang et al. reported the NIR fluorescent probe DCM-Br-ONOO (Figure 12d), which can be localized in the endoplasmic reticulum and respond to endogenous ONOO^−^ in A549 cells [84]. The probe comprises two components: the fluorescent group DCM-Br and the recognition group pentafluorobenzenesulfonate. The pentafluorobenzenesulfonate in the probe possesses electron-absorbing characteristics, inhibiting the ICT process of DCM-Br and maintaining the probe’s initial fluorescence in the off state. Upon entering the cell, the probe spontaneously localizes in the endoplasmic reticulum due to its lipid-soluble properties. During this process, ONOO^−^ attacks the pentafluorobenzenesulfonate group as a nucleophilic reagent, triggering a cleavage reaction that releases the DCM-Br fluorescent group and activates the ICT process. Specifically, electron transfer from the donor (oxygen atom) to the acceptor (DCM) results in fluorescence activation. Consequently, the level of ONOO^−^ can be imaged and quantified based on the intensity of the fluorescence signal. Additionally, the team innovatively incorporated Br into the fluorescent group to modify the *pKa* value of the probe, thereby extending the physiological pH range for imaging. This study offers novel insights and directions for investigating the interaction between ONOO^−^, endoplasmic reticulum stress (ERS), and various diseases.

**Figure 13:**
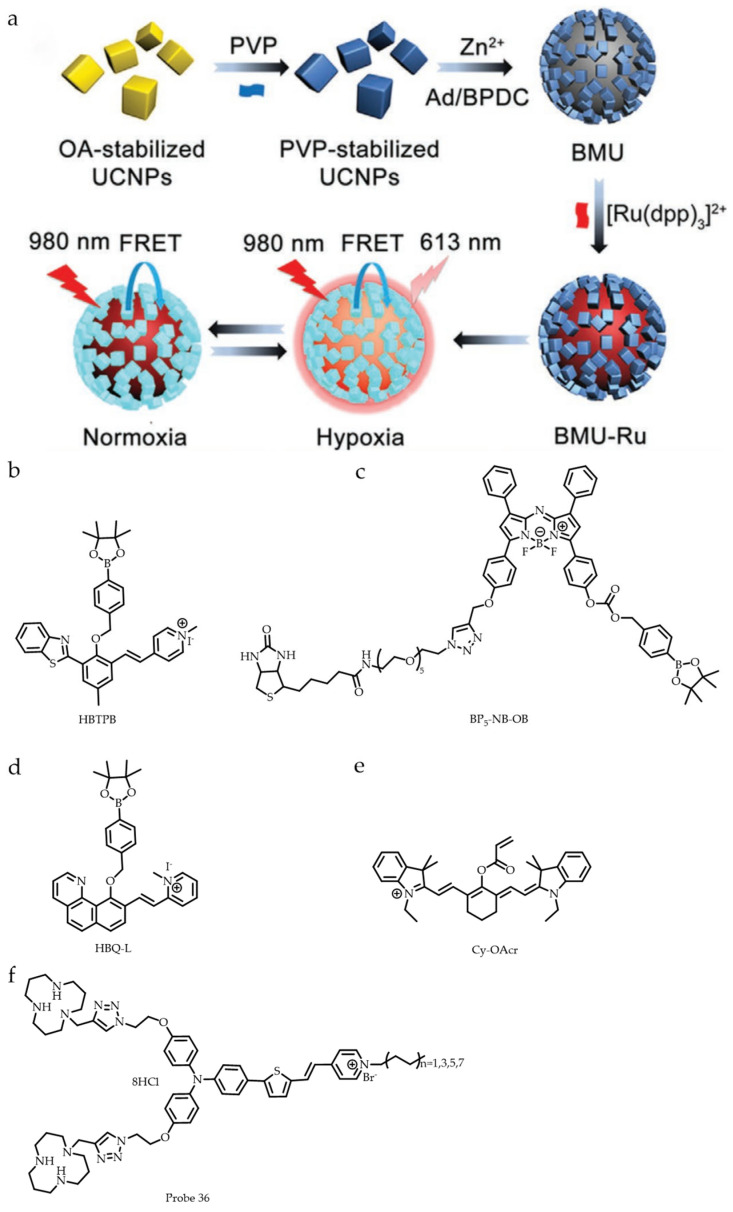
(**a**) The construction process and functional principle of BMU-Ru, reproduced from [85] with permission from John Wiley and Sons, Copyright 2020; (**b**) Structure of HBTPB; (**c**) Structure of BP_5_-NB-OB; (**d**) Structure of HBQ-L; (**e**) Structure of Cy-OAcr; (**f**) Structure of Probe 36 (gene vector with AIE characteristics).

Oxygen concentration is a critical parameter of the tumor microenvironment and is closely associated with the occurrence, development, prognosis, and treatment of NSCLC. Monitoring oxygen concentration is crucial for the diagnosis, treatment, and prognosis assessment of lung cancer. In 2020, Li et al. reported the development of an NIR excited oxygen concentration detection nanosensor, BMU-Ru (Figure 13a), for monitoring the progression of NSCLC lesions [85]. The team chose tris(4,7-diphenyl-1,10-phenanthroline) ruthenium(II) dichloride ([Ru(dpp)_3_]^2+^Cl_2_) as the oxygen sensor for BMU-Ru, which exhibits fluorescence intensity variations in response to changes in oxygen concentration. By integrating the oxygen sensor with upconversion nanoparticles (UCNPs), energy is transferred to [Ru(dpp)_3_]^2+^Cl_2_ via Förster resonance energy transfer (FRET) upon excitation with 980 nm NIR light, enabling the indirect monitoring of oxygen concentration through the fluorescence intensity of [Ru(dpp)_3_]^2+^Cl_2_. The successful application of BMU-Ru in mouse models of NSCLC tumors further demonstrates its capability to monitor the progression of NSCLC lesions.

Hydrogen peroxide (H_2_O_2_) plays a crucial role in various physiological processes. Abnormal production and excessive accumulation of H_2_O_2_ are significant contributors to various diseases, including tumors. Mitochondria are primary sources of H_2_O_2_; thus, monitoring H_2_O_2_ levels in mitochondria within living cells is of paramount significance. In 2018, Tang et al. developed the ratiometric fluorescent probe HBTPB (Figure 13b), which can localize in mitochondria and respond to endogenous H_2_O_2_ in A549 cells [86]. The probe’s mechanism of action is based on excited state intramolecular proton transfer (ESIPT) and the specific reaction of phenylboronate with H_2_O_2_. As a nucleophilic reagent, H_2_O_2_ reacted with the phenylboronate group to form a phenolic hydroxyl group. The release of the phenolic hydroxyl group eliminates the inhibitory effect of ESIPT, resulting in a transition of the probe from a non-fluorescent state to a fluorescent state. Furthermore, the fluorescence intensity of the probe varies with changes in H_2_O_2_ concentration. As the H_2_O_2_ concentration increases, the fluorescence intensity of the probe at 539 nm gradually decreases, while that at 669 nm gradually increases, ultimately forming a new fluorescence emission peak. In 2019, Mao et al. utilized the ICT effect and BODIPY derivatives to develop another ratiometric fluorescent probe, BP_5_-NB-OB (Figure 13c), achieving the in vivo imaging of tumors by detecting endogenous H_2_O_2_ in the A549 mouse model [87]. The probe detects H_2_O_2_ by modulating the ICT process. The carbonyl group in the probe serves as an electron-deficient linker that blocks the ICT process, maintaining the probe in a non-fluorescent state. In the presence of H_2_O_2_, it attacks the pinacol borate group in the probe, leading to its cleavage and the generation of NB-OH, which possesses stronger electron donor capabilities. This allows for an ICT process with the carbonyl group, resulting in a red-shift of the probe’s absorption and emission spectra and generating a ratiometric NIR fluorescence signal. In 2022, Chen et al. also employed the ESIPT principle to develop a fluorescent probe named HBQ-L (Figure 13d), achieving high sensitivity and specificity in detecting H_2_O_2_ in zebrafish and tumor mouse models [88]. The HBQ in the probe is linked to the benzyl boronic pinacol ester group via pyridine. The pyridine moiety in the probe facilitates its targeting to the mitochondria, as it can interact with the negative charge on the mitochondrial membrane. Simultaneously, the HBQ structure exhibits ESIPT activity. In the excited state, protons are transferred within the HBQ structure, resulting in the generation of two distinct fluorescence emission peaks. Following the probe’s reaction with H_2_O_2_, the fluorescence emission peak red-shifts from 508 nm to 642 nm. This enables the quantitative detection of H_2_O_2_ concentration by measuring the fluorescence signal intensity ratio of I642/I508. These fluorescent probes offer novel insights and perspectives for investigating the mechanisms of H_2_O_2_ action and disease, as well as for the in vivo imaging of lung cancer.

Cysteine (Cys) is a sulfur-containing amino acid with sulfur atoms in its side chain. It is a crucial biological thiol with various essential roles in living organisms and primarily exists in the reduced state (-SH groups) within the body. Cysteine can be oxidized to form cystine, where two cysteine molecules are linked by a disulfide bond. Elevated levels of Cys in tumor tissues compared to adjacent normal tissues suggest that Cys may serve as a potential biomarker for tumors. In 2019, Zhang et al. reported an NIR fluorescence probe, Cy-OAcr (Figure 13e), designed to assess Cys levels in an in situ lung cancer model [89]. The probe utilizes the acrylate group as the Cys-responsive element, while the lipophilic iminium cation unit facilitates mitochondrial targeting. Cy-OAcr effectively detected and imaged Cys in three tumor cell lines and their corresponding tumor-bearing mouse models. It distinguished tumor tissue from normal tissue based on Cys levels in an in situ lung cancer model, demonstrating its significant potential for clinical cancer diagnosis.

As previously mentioned, Li et al. integrated gene therapy into a multifunctional nanoparticle in 2020. In the following year, Tang et al. reported a gene vector (Figure 13f) (labeled as Probe 36 in this article), with AIE characteristics capable of non-viral gene delivery, gene delivery process tracking, in vivo imaging, and cancer therapy [90]. Additionally, they incorporated photodynamic therapy into the carrier, resulting in a synergistic effect between gene therapy and photodynamic therapy, which significantly enhanced tumor treatment efficacy. The team developed an NIR luminous core with a typical D-π-A structure, which endowed the carrier with favorable optical properties and the capability to produce singlet oxygen (^1^O_2_). The two polar aneN3 groups exhibit a strong affinity for negatively charged nucleic acids, facilitating the condensation of loose nucleic acids into positively charged nanoparticles and enhancing cell binding and endocytosis. The hydrophobic tail, composed of long carbon chains, enhances the fusion of the carrier with the cell membrane, thereby facilitating endosome escape. By combining the vectors with DNA fragments capable of gene therapy to form vector/DNA complexes, the carrier/DNA complex demonstrated low cytotoxicity, high gene transfection efficiency, good biosafety, and effective tumor targeting in experimental models involving A549 cells and tumor-bearing mice. This complex holds promising prospects for application in cancer therapy and bioimaging.

The probes summarized in this section complement the first four types of probes to some extent. Although not categorized as any of the first four types, they still demonstrate unique advantages in lung cancer diagnosis and treatment. These fluorescent probes, associated with organelles, active substances, and cell metabolites, are crucial for studying the role of these biomarkers in lung cancer progression. Moreover, their importance extends beyond lung cancer, as numerous studies indicate that these biomarkers are closely linked to the occurrence and development of other tumors and even various diseases. This knowledge could serve as a valuable reference for developing more advanced fluorescent probes for lung cancer diagnosis in the future.

Upon completing the summary of probes in this chapter, we believe readers will gain a deeper understanding of the current development status of NIR fluorescent probes in lung cancer through this classification. In the following chapters, we will also provide a brief summary of the clinical applications of current fluorescent probes. Additionally, we will discuss the future development directions and current bottlenecks in NIR fluorescence probe development. To facilitate a more intuitive understanding of these NIR fluorescent probes, we have summarized their characteristics in Table 1, organized according to the order described in this chapter.

## 4. Clinical Application and Transformation

Several fluorescent probes currently on the market have received regulatory approval for clinical in vivo detection. Notably, indocyanine green (ICG) has been sanctioned by regulatory bodies such as China’s National Drug Administration, the FDA, PMDA, and EMA and is widely used for detecting various diseases. In 2016, Kim et al. assessed the safety and feasibility of using low-dose ICG for intraoperative detection of lung tumors through NIR fluorescence imaging [91]. Their clinical study involving 11 patients undergoing lung tumor resection demonstrated that NIR fluorescence imaging effectively identified lung tumors without adverse effects. The same year, Keating et al. evaluated OLT38, an ICG-based fluorescent probe, for its ability to locate primary lung adenocarcinoma, perform lymph node sampling, and assess tumor margins [92]. The fluorescence patterns obtained with OLT38 correlated well with pathological findings and clearly delineated tumor boundaries, confirming its utility in the fluorescence imaging of human lung cancer. Furthermore, in 2019, Newton highlighted that the optimal ICG dose for NIR imaging varies with tumor histology: lower doses (2–3 mg/kg) are more effective for non-primary lung cancer, whereas higher doses (4–5 mg/kg) are preferred for primary lung cancer [93]. Recent advancements have led to the development of various fluorescent probes based on ICG, each offering unique advantages in their respective applications. Among these, Cytalux has demonstrated particular promise and received FDA approval in 2021 for ovarian cancer detection and surgical imaging, providing significant support for both surgeons and ovarian cancer patients.

In addition to the approved fluorescent probes, some probes are currently in the clinical trial stage. One representative is the broad-spectrum tumor diagnostic probe NC527-X (https://www.cde.org.cn/main/xxgk/listpage/9f9c74c73e0f8f56a8bfbc646055026d; accessed on 10 October 2024; acceptance inquiry number: CXHL2400661). However, due to commercial confidentiality, specific information about NC527-X remains unavailable. According to current data, it can perform the in vivo imaging of various tumors, including lung cancer. NC527-X specifically targets tumor cells and, when combined with popular NIR fluorescence imaging technology, achieves non-invasive, deep, high-definition, and sensitive imaging of tumors. This also highlights the significant application potential of NC527-X in tumor diagnosis, precision treatment, and other areas.

At present, there are many fluorescent probes currently undergoing clinical trials. To offer readers a clearer understanding, we have summarized the fluorescence imaging probes in the clinical stage in Table 2. To include a comprehensive list of fluorescent probes relevant to lung cancer, non-NIR probes are also included in the table.

In 2018, Newton et al. explored the potential applications of intraoperative fluorescence imaging in cancer surgery, based on existing preclinical and clinical data [98]. Their work highlighted the broad prospects of fluorescence imaging technology for thoracic malignant tumor surgeries. They posited that advancements and the application of new fluorescent dyes could enhance the accuracy and safety of surgical procedures in the future. In 2020, Jiao et al. reviewed recent developments in tumor-targeted NIR fluorescence dyes and their molecular imaging applications in clinical trials and preclinical studies [99]. They concluded that these dyes hold significant promise for fluorescence-guided surgery, potentially improving tumor resection rates and patient outcomes. In 2022, Neijenhuis et al. examined the use of NIR fluorescence imaging for the localization and identification of tumors during lung cancer surgeries [100]. They argued that while NIR fluorescence imaging shows considerable potential for intraoperative tumor localization and identification in lung cancer, further clinical studies are needed to confirm its effectiveness and safety. In addition to the aforementioned studies, NIR fluorescent probes utilized for precise intraoperative navigation have also been reported in the past two years. For instance, Chen et al. reported in 2024 the NIR fluorescent probe NIR-LD, designed for the precise imaging of lipid droplets in tumors [101]. This probe can monitor changes in the polarity, size, and number of lipid droplets, as well as dynamic behaviors such as fusion, fission, transfer, growth, and lipolysis, thereby providing a powerful imaging tool for NIR fluorescence navigation during surgery. In 2024, Xiang et al. employed the latest machine-learning algorithms to assist in the design of new fluorescent molecules, resulting in the development of the SiRCTS-pH probe [102]. The team utilized this probe to conduct surgical navigation experiments in a hepatocellular carcinoma mouse model, successfully achieving precise tumor positioning. Additionally, similar work was reported in 2023, including the J-S-LS301 probe developed by Zhang et al., which exhibits strong specificity and targeted fluorescent tumor imaging capabilities [103]. This probe has been validated in both in situ transplanted liver cancer models and multi-nodule in situ transplanted liver cancer models, demonstrating significant potential in the biomedical field and its applicability for fluorescent tumor navigation surgery. In 2023, Zhang et al. also developed the NIR fluorescent probe Si-NH2-Glu based on γ-glutamyl transpeptidase (GGT), which can specifically identify tumor cells and realize fluorescent imaging of the edge of breast cancer [104]. It has good potential for application in intraoperative navigation and provides new possibilities for precision surgery; Ran et al. reported a multifunctional NIR-II fluorescent probe called ICR-QuNPs, which has a high photothermal conversion efficiency, excellent NIR second-zone fluorescence emission, and multimodal imaging capabilities [105]. Its excellent performance enables it to be used in multimodal imaging-guided photoimmunotherapy and intraoperative navigation. These studies offer a robust theoretical foundation for NIR fluorescence surgical navigation and tumor resection, while also serving as a valuable reference for the future development of NIR fluorescence imaging technology.

Although the clinical translation of NIR fluorescence imaging probes is a prominent research focus, the process remains challenging. As noted, Cytalux’s (On Target Laboratories, a biotechnology company based in West Lafayette, USA) predecessor, OTL38, was reported by Dutch scientists as early as 2011, yet it took ten years for Cytalux to receive official FDA approval. To accelerate clinical translation, scientists are exploring innovative approaches, such as developing new fluorescent probes that combine clinically approved targeted ligands with fluorescent dyes proven to be non-toxic in vivo. For instance, Pal et al. reported a clinical trial in 2022 involving the probe Panitumumab-IRdye800CW (LI-COR Biosciences, a biotechnology company based in Lincoln, USA), which uses the FDA-approved EGFR therapeutic antibody panitumumab as the targeting component [106]. IRdye800CW, previously tested in multiple human trials, has demonstrated minimal biological toxicity, making it an ideal NIR fluorescent dye. The clinical trial results indicated that the probe has a favorable safety profile in patients with head and neck squamous cell carcinoma (OSCC).

In addition to simple detection imaging, many researchers are working on integrating therapeutic functions into fluorescent probes. For instance, Lucero et al. (2021) designed a PARx prodrug for lung cancer treatment while also developing a diagnostic probe, PACDx [54]. Similarly, Barth et al. (2022) synthesized a chemo-cat DOX alongside probes for tumor diagnosis, where doxorubicin attached to the probes selectively targets and kills tumor cells without affecting normal cells [107]. Photodynamic therapy is another highly effective method. Drawing inspiration from the NETosis process, Gong et al. (2023) developed nucleotide networks (FNATs) that mimic NETs for precise tumor cell recognition [108]. Once tumor cells are identified and an ordered FNATs network is formed, nucleic acid probes containing Ce6 photosensitizing agents can be captured and used to precisely kill tumor cells upon light activation.

In 2021, Liu et al. reviewed current clinical translational trends and key considerations regarding fluorescent probes in surgical navigation [109]. They highlighted the significant potential of fluorescent probes for surgical navigation but noted that several key issues need to be addressed. These include improving the signal-to-noise ratio, reducing background signal interference, ensuring safety, and developing reliable manufacturing processes. Additionally, Liu et al. anticipated that NIR-II fluorescence imaging would see increased application in the future. Similarly, Yang et al. (2021) reviewed the use of NIR-II fluorescence imaging in tumor surgical navigation, noting its advantages due to lower tissue scattering, absorption, and endogenous fluorescence [13]. This results in deeper tissue penetration and higher imaging resolution compared to NIR-I imaging. They also observed that existing NIR-I fluorescent dyes, such as ICG and IRDye800CW, exhibit longer emission tails in the NIR-II region, making them useful as a transitional step towards NIR-II fluorescence imaging. In a 2023 study, Mi et al. compared the efficacy of NIR-I and NIR-II fluorescence imaging in lung cancer surgery by conducting clinical trials involving 102 patients scheduled for lung cancer surgery [110]. Their findings corroborated Yang’s conclusions, demonstrating that NIR-II fluorescence imaging offers significant advantages over NIR-I fluorescence imaging for surgical navigation.

## 5. Conclusions and Perspectives

In the ongoing pursuit of improved methodologies, scientists have developed increasingly advanced detection technologies and treatment methods by analyzing the advantages and limitations of existing technologies and integrating contemporary advancements. Fluorescent probes have emerged as a prominent area of research in tumor detection and treatment, offering broad application prospects. Although fluorescent probes have gained significant attention in tumor diagnosis and treatment due to their unique advantages and substantial progress, several challenges remain in the field of fluorescence probe technology.

Optimization of Design Strategy: High sensitivity and selectivity are essential for fluorescent probes to accurately detect low-concentration tumor markers in early cancer diagnosis, necessitating the exploration of optimized design strategies to enhance the signal-to-noise ratio (SNR) and sensitivity of these probes. Specifically, biomarker-activated probes can be combined with VEGF-targeted probes to enhance their targeting capabilities. Additionally, the work by He et al. (2021) [111] and Zhang et al. (2022) [112] can serve as a reference, where they developed multi-target cascade-activated probes to enhance identification accuracy. Furthermore, combining the strengths of various probe types to develop a multi-type detection system is worth considering. For example, the LFIA [65] platform developed by Li, mentioned earlier, integrates enzyme and antigen probes, thereby reducing the likelihood of misdiagnosis and missed diagnoses.

Slow Progress in Clinical Translation: Despite notable advancements in NIR imaging, the clinical translation of fluorescent probes has been limited. This delay is attributed to three primary factors: (i) The development of NIR fluorescent dyes: Dyes must exhibit strong fluorescence signals, a high signal-to-background ratio, and resistance to photobleaching, yet few dyes meet these criteria for clinical NIR imaging. (ii) Selection of targets: Fluorescent probes must selectively bind to target ligands, such as tumor-related markers, in diseased tissues to ensure adequate fluorescence intensity at the target site. (iii) Hardware and software development: Clinical imaging equipment must be highly sensitive, capable of detecting NIR wavelengths, and designed with ergonomic features for practical clinical use. Currently, indocyanine green (ICG) is the predominant NIR fluorophore used in clinical settings, leading to a predominance of imaging devices compatible with ICG and fewer options for other fluorophores.

Molecular Size and Hydrophilicity: The molecular size and hydrophilicity of the molecular probe significantly influences its residence time within internal blood vessels and extravascular lumens. Smaller molecular probes, typically more permeable and metabolizable, are often better suited for localized applications, whereas larger probes struggle to penetrate deeper into tissues. Current strategies to enhance retention time involve designing biodegradable probes that decompose into smaller fragments, which can then be excreted by the kidneys.

Functional Integration: The current focus in fluorescent probe development should be on creating multifunctional probes that integrate additional capabilities beyond basic imaging. These capabilities may include targeted tumor treatment with chemotherapy drugs and photosensitizers, real-time monitoring of drug release and treatment efficacy, enhancement of drug resistance in specific tumor types, and incorporation of immunotherapy concepts to activate the body’s immune system for tumor metastasis inhibition.

Interdisciplinary Collaboration: To address the current challenges in fluorescent probe development, interdisciplinary cooperation is essential. Chemists and chemical engineers are developing more suitable fluorophore compounds for tumor detection; clinical researchers are identifying additional tumor markers to design more specific probes; physicists, engineers, and electronics experts are creating imaging devices that can accommodate various fluorophores or respond to multiple fluorophores across different wavelengths. Furthermore, artificial intelligence researchers are applying advanced technologies to enhance probe design, device development, and detection algorithms. Overall, a collaborative, multidisciplinary approach is crucial for advancing fluorescent probe technology.

For lung cancer, a key consideration is the unique anatomical environment of the lungs within the human body. Multiple organs overlap in the chest cavity, and there are certain spaces around the lung organs, which demands an enhanced tissue penetration capability from imaging probes. Therefore, the longer emission wavelength of NIR-II probes currently presents a more effective solution. Another approach is to combine NIR fluorescence imaging technology with photoacoustic technology to develop a multimodal imaging tool, which can significantly enhance the effectiveness of in vivo lung cancer imaging. Given the current emphasis on fluorescence navigation in surgery, which requires imaging tools with extremely high specificity, sensitivity, and targeting ability, it is crucial to further improve these three aspects of NIR fluorescence imaging tools. Additionally, using in vitro diagnostic tools to complement in vivo imaging tools is essential for accurately assessing the progression of lung cancer in patients. In vitro diagnosis is not constrained by the physiological environment of the body and requires only the ability to accurately and efficiently diagnose lung cancer. Therefore, pleural effusion, blood, urine, and other accessible samples can be utilized as diagnostic materials. Taking the antigens, enzymes, nucleic acid fragments, and other biomarkers highly related to lung cancer in these samples as a collection, it is a promising direction for scientists to develop NIR fluorescence probes that can simultaneously respond to these markers.

In general, lung cancer remains a severe disease with a high fatality rate. Currently, NIR imaging is a promising research area, demonstrating capabilities in various aspects of disease diagnosis and treatment. NIR imaging technology plays a crucial role in the detection, diagnosis, treatment, and prognosis of lung cancer. With continued efforts, it is anticipated that a variety of high-performance, multifunctional, and low-cost NIR fluorescence imaging technologies will be available in the future.

## Figures and Tables

**Table 1:** Summary of general characteristics of NIR fluorescent probes.

Serial Number	Name	λ_ex_ (nm)	λ_em_ (nm)	Stokes Shift (nm)	Treatment Type	Species of Detection	Cell Localization
01	GNS@BSA/I-MMP2	710	790	80	PTT/PDT	MMP-2	Cytoplasm
02	F-1	468	650	182	-	NE	-
03	DMC-βgal	725	770	45	-	β-gal	-
04	HA-apn	550	835	285	-	APN	Cytoplasm
05	ENBO-ML210	640 ± 20	700 ± 20	±60	-	GPX4	Mitochondria/Lysosomes/Endoplasmic reticulum
06	NIR-ASM	460	646	186	-	NQO1	Cytoplasm
07	LET-10	660	740	80	-	NQO1	-
08	HCy-Q	680	747	67	-	NQO1/Viscosity	Mitochondria
09	HD-F	635	660–750	25–115	-	Furin	Golgi apparatus
10	TTPI	-	690	-	PDT/Chemotherapy	COX-2	Golgi apparatus
11	IR-ABS	725	850	125	-	NADH/NTR	-
12	IR-780-Crizotinib	-	809	-	Chemotherapy	ALK	-
13	TPG	635	670–770	35–135	Chemotherapy	GSH	Cytoplasm
14	CEL@G-SS-NIR	675	710	35	Chemotherapy	GSH	-
15	TBG	704	690–800	−14–96	Chemotherapy	GSH	Cytoplasm
16	CY3-AFTN and Cy5-AFTN	488/641	570/674	82 and 33	-	HER1/HER2	Endoplasmic reticulum/Mitochondria/Nucleus
17	LX	720	750	50	-	ROS	-
18	NIR-CDs-DNA-AuNRs@SiO2-Aptamer	420	683(NIR-CDs)	263	-	CEA	Nonvivo probe
19	Probe 19	980	540	−440	-	CYFRA21-1	Nonvivo probe
20	Probe 20	780	1050	270	-	CEA/CYFRA21-1/NSE	Nonvivo probe
21	Probe 21	-	626	-	-	CEA/NSE	Nonvivo probe
22	pH-AAP	640	650–720	10–80	-	Acidic environment	Membrane/Lysosome
23	CE7Q/CQ/S	633	806	173	Chemotherapy/Gene therapy/PTT	Acidic environment	Lysosome
24	PMT9	-	-	-	-	Acidic environment	-
25	BDHT	561	663–738	102–177	-	Viscosity	LDs
26	BFZ	633	650–750	17–117	-	Polarity	LDs
27	rova-IR700	-	-	-	-	DLL3	Membrane
28	Cy-Lyso	760	782	22	-	Organelle	Lysosome
29	IR-34	770	830	60	Paraptosis	NDUFS1	Mitochondria
30	DCM-Br-ONOO	570	680	110	-	ONOO^−^	Endoplasmic reticulum
31	BMU-Ru	980	613	−367	-	Oxygen concentration	-
32	HBTPB	312	669	357	-	H_2_O_2_	Mitochondria
33	BP_5_-NB-OB	675	700/720	25–45	-	H_2_O_2_	-
34	HBQ-L	405	642	237	-	H_2_O_2_	Mitochondria
35	Cy-OAcr	520/550/740	-	-	-	Cys	Mitochondria
36	Probe 36	504 (one-photon) 1040 (TP)	550/870	-	PDT/Gene therapy	-	Cytoplasm

**Table 2:** Fluorescent probes in the clinical stage.

Serial Number	Name	NIR-1/II	Target	Route of Administration	Cancer Type	Clinical Status (I/II/III)
01	EC-17 [94]	Non-NIR	Folate receptor	Intravenous	Lung, breast, ovarian, renal cell cancer	I/II
02	GE-137 (EMI-137) [95]	NIR-I	c-MET	Intravenous	Lung, colonic cancer	I/II
03	SGM-101 [96]	NIR-I	CEA	Intravenous	Lung, colon, rectum, pancreas cancer/Peritoneal carcinomatosis/Colorectal neoplasms	I/II/III
04	Panitumumab-IRDye800CW [97]	NIR-I	EGFR	Intravenous	Lung carcinoma/Brain tumor/Pancreatic cancer	I/II
05	OTL-38 [12]	NIR-I	Folate receptor	Intravenous	Lung, ovarian cancer	III
